# Management of Acute Abscesses by Day-Case Surgery: A Retrospective Analysis and Feasibility Study

**DOI:** 10.7759/cureus.75568

**Published:** 2024-12-11

**Authors:** Lewis Blenkinsop, Jason Ramsingh

**Affiliations:** 1 General Surgery, Newcastle Upon Tyne Hospitals NHS Foundation Trust, Newcastle, GBR

**Keywords:** abscess, abscess pathway, complications, day case, day-case, day surgery, drainage, inicison and drainage, quality improvement

## Abstract

The management of acute abscesses is a common problem that is faced by surgical departments around the world. Once the decision is made to proceed to surgical drainage, allocating patients to an operative list can lead to delays and unnecessary admission to hospital overnight. The British Association of Day Surgery recognises acute abscess as a condition that can be managed in Day Surgery Centres.

We retrospectively reviewed 100 abscess incision and drainages in our University Teaching Hospitals’ Acute General Surgery department to see what parameters would make a patient suitable to undergo this procedure as a day-case surgery. This analysis was performed in the hope of implementing a new pathway for their care to streamline the emergency operating list. A total of 83 patients were suitable to be included in the analysis.

Of these 83 cases, 71 patients were discharged on the day of surgery, giving us an 85.5% day-case rate. We found that pilonidal and trunk abscesses had a 100% (23 out of 23 cases) day-case rate while patients who also suffered from diabetes had only a 71.4% day-case rate (10 out of 14 cases). The rate of overnight admission increased as American Society of Anesthesiologists (ASA) score increased and perianal abscesses while being the most frequently operated upon, had only a 76.9% day of surgery discharge rate (20 out of 26 cases).

We felt reassured by the research that with proper patient selection a large percentage of our abscesses could be managed in a Day Surgery Unit and we have begun implementation of this pathway. We hope it can reduce unnecessary hospital admissions, reduce pressures on emergency theatres and prevent patients from waiting prolonged periods of time for an urgent operation.

## Introduction

Acute abscesses are a common problem encountered in hospital medicine and primary care. In 2005 there were over three million attendances to the emergency department in the US due to cutaneous abscesses [1] and the management of these in the United Kingdom usually falls under the care of an acute surgical team. These cutaneous abscesses can occur almost anywhere in the body and each location carries its own risks and complications [2]. Many surgical departments will have their own pathways for managing these abscesses and the clinical need to perform drainage depends on location, clinical condition and patient comorbidities. Once it is agreed that an abscess requires drainage further consideration must be given to whether this is to be performed under a general or local anaesthetic.

In our trust patients who required drainage under a general anaesthetic but were not acutely septic would be advised to fast from midnight and return the next morning for review. We would then aim to operate on them first on the emergency list that day. We found that we were occasionally having issues with there being too many patients with abscesses to operate on in one day and considered if it was possible to schedule some of these patients onto a day-case list. The National Day Surgery Delivery Pack [3] lists acute abscess as a condition that could be managed in a day-case setting and also states that Day Surgery should be the default option for the procedures it lists.

The Royal College of Anaesthetists states that 2%-10% of Day Surgery cases may require unplanned admission after their procedure depending on the complexity of the operation. It lists the recommended percentage of emergency incision and drainages achievable as day cases to be 100%. This means that we need to aim for 98% of abscess drainages being discharged the same day or a <2% unplanned admission rate [4].

While other studies have demonstrated the cost-effectiveness of moving to an ambulatory approach of abscess incision and drainage [5], we wanted to assess what patients would actually be eligible before setting up a service. With this target in mind we began a retrospective study to see which of our abscesses could be safely managed in a Day Surgery setting in the hopes of creating a new pathway for these patients to ease the clinical burden on emergency theatres, prevent delays to patient care and reduce unnecessary admissions.

## Materials and methods

We searched our emergency theatre lists for 100 incision and drainages performed in emergency theatres between 01/08/2022 and 10/11/2022. We wanted only to include patients who would have been eligible for outpatient, day-case management of their abscess by the general surgery team. This meant the operation had to have occurred in hours (started between 0800-1700), that the patient had to have been operated on by general surgery on the same day as admission and that the patient had to be systemically well with no pyrexia or tachycardia.

Of the 100 cases, 91 were admitted via the acute surgical team on the same day as the operation. Eight of these however had their operation out of hours without any clear indication as to why an out of hours operation was required (no documented indication, no pyrexia or tachycardia). There are a variety of reasons as to why the team may have decided this, such as if a patient has been waiting all day and a desire not to delay an operation a further 24 hours with no guarantee of theatre space the next morning. Due to the delay in beginning their operation, these eight remained inpatient overnight despite there being no obvious clinical need documented. This leaves us with 83 patients who were operated on in hours, the same day as their initial assessment by the surgical team and who were systemically well, thus suitable for an ambulatory abscess service. 

We reviewed the medical history of these patients to look for any comorbidities that may complicate the procedure. We reviewed their clinical condition and documented history of presenting complaint as acutely unwell patients would not be eligible for day-case management. We also reviewed the operation notes to confirm the location of the abscess.

## Results

A total of 83 cases were included in the full analysis. The average age of these patients was 40.3 years (range of 17-79 years). 16.9% of patients (14 patients) suffered with diabetes. 79.5% of total cases (66 patients) were American Society of Anesthesiologists (ASA) I or II. The location of abscess varied as shown in Table 1. The most common site was perianal comprising 31% of cases (26 patients) followed by pilonidal 17% (14 patients) and then groin with 15% of cases (12 patients). The data spanned 101 days with abscess incision and drainage occurring on 60 of these days. Including the outliers removed previously, there were 10 days where three or more abscesses were drained in the emergency theatre. Six of the eight cases removed for being operated on out of hours occurred on these days with three or more incision and drainages. 

**Table 1:** Showing abscess location and rate of day of surgery discharge

Abscess Location	Number of Cases	Number Day Case	Percentage Day Case
Abdominal Wall	4	4	100.0%
Axilla	10	9	90.0%
Back	5	5	100.0%
Buttock	6	5	83.3%
Groin	12	9	75.0%
Neck	2	2	100.0%
Perianal	26	20	76.9%
Pilonidal	14	14	100.0%
Shoulder	3	2	66.7%
Thigh	1	1	100.0%
Total	83	71	85.5%

Of the 83 cases 71 were completed as a day-case, giving an overall day-case rate of 85.5%. 

The average age of patients who were discharged on the same day as surgery was 38.8 years (range of 17-79 years), in patients who were not discharged on the same day this age increased to 47.5 years (range of 31-79 years).

Of the 14 patients with diabetes 71.4% were discharged the same day (10 patients). That means that 33.3% of patients who remained overnight (four of the 12 patients) were diabetic.

As ASA increased the rate of day of surgery discharge decreased. Thirty-one patients were ASA I and of these 93.5% (29 patients) were discharged the same day. Thirty-five patients were ASA II and of these 82.9% (29 patients) were discharged the same day. Seventeen patients were ASA III or greater and 76.5% (13 patients) were discharged on the same day. 

The most common comorbidity among the patient group was diabetes.

Factors affecting day of surgery discharge were as described above. ASA I and II patients had a combined day of surgery discharge rate of 86.6% (58 out of 67 cases). One hundred percent of trunk and pilonidal incision and drainages were day-case (25 cases) but only 75% of groin (nine out of 12 cases) and 76.9% of perianals (26 cases). Of the two patients taking blood thinners one was discharged the same day.

## Discussion

This study shows the feasibility of day case surgery for acute abscess if the patient selection is careful and the patients are clinically stable enough to have surgery delayed until the next day case list. In particular, the location of the abscess appears important, with pilonidal abscesses appearing the easiest to complete in an expedited day-case manner. If aiming for >98% of patients being discharged on the day of surgery this study would suggest that pilonidal and trunk abscesses can be performed in a Day Surgery setting. Abscesses of the axilla may be eligible but more data would be required.

It also highlights the difficulty in operating on diabetic patients in an ambulatory setting with 28.6% of these patients requiring admission overnight (four of the 14), suggesting they would not be eligible for such a pathway. 

Other factors are at play than just abscess location and diabetic status. There is a trend with ASA grades suggesting only patients with an ASA I or II should be offered ambulatory drainage. This would be contrary to other studies that have found that ASA III patients have no increased rate of unplanned admission following day surgery [6]. This may be due to the nature of this still being an emergency surgery with no time to prehabilitate the patient. An exception is made in the National Day Surgery Delivery Pack for guidance regarding a patient's ASA, as it accepts patients with a higher ASA may require an inpatient stay for urgent procedures [3]. This is an example of where typical guidance for elective day surgery does not apply to urgent or emergency day surgery cases.

Further study into patients on blood thinners would be required as only two were included in this data set.

There is a national target for 85% of operations to be conducted in a day-case setting [3] so it is important to assess what procedures and patients are eligible to be managed in this way. The benefits of day-case surgery are numerous. Previous studies looking at abscess management have detailed the financial case [5,7] but there are also significant benefits to the patient experience with patients who undergo day-case surgery generally going on to have a preference for this type of surgery in the future [8,9]. 

In our initial data set of 100 procedures, eight patients had their operation out of hours and a subsequent overnight admission with no clear documentation as to why admission or urgent operative management of the abscess was necessary. In six of these cases, three or more abscesses were drained on the same day perhaps indicating the clinical burden on these days. It would be understandable if a patient has waited and remained fasted since 0800 that there would be a desire to continue operating out of hours to ensure their procedure was completed rather than delayed indefinitely. However, due to the delay in starting their case, they likely had an unnecessary admission to hospital. In cases like these if admission could be avoided benefits would not just be financial and of patient experience but also reduce bed pressures in the hospital. This is an important consideration globally but particularly in the UK where we have “fewer hospital beds to comparable nations” [10].

While the study has helped to identify which patients are likely to achieve day of surgery discharge, it does not help regarding the selection of patients that are suitable for ambulatory management of their abscess. In Olugbemi et al. [5] they discussed their use of blood tests to guide suitability for ambulatory management. This is not routine practice in our department where we depend more upon clinical examination and patient history. As discussed above all of the selected patients had normal observations on initial assessment but ultimately the clinical decision that an operation could be delayed would rest with the surgeon reviewing the patient. This is an additional reason why patients with diabetes would be unlikely to be eligible for ambulatory management as multiple studies have demonstrated the increased risk that infection poses to patients who suffer from diabetes. These patients have up to a five times increased risk of complication due to a skin and soft tissue infection [11] and their risk of death due to an infection is almost doubled [12].

## Conclusions

The study has shown that day-case abscess drainage is feasible and built upon previous studies demonstrating the cost-effectiveness of day-case surgery. It has shown that there are a variety of factors to consider as well as the usual patient factors suggested in the The National Day Surgery Delivery Pack. Based on this study we will begin implementing an ambulatory abscess pathway in our department. We will monitor the success of day-case abscess drainage, aiming for a <2% unplanned admission rate as suggested by the Royal College of Anaesthetists. We hope this can free up emergency operating space, prevent unnecessary overnight admissions and reduce the delays in patients receiving the operation they need.
